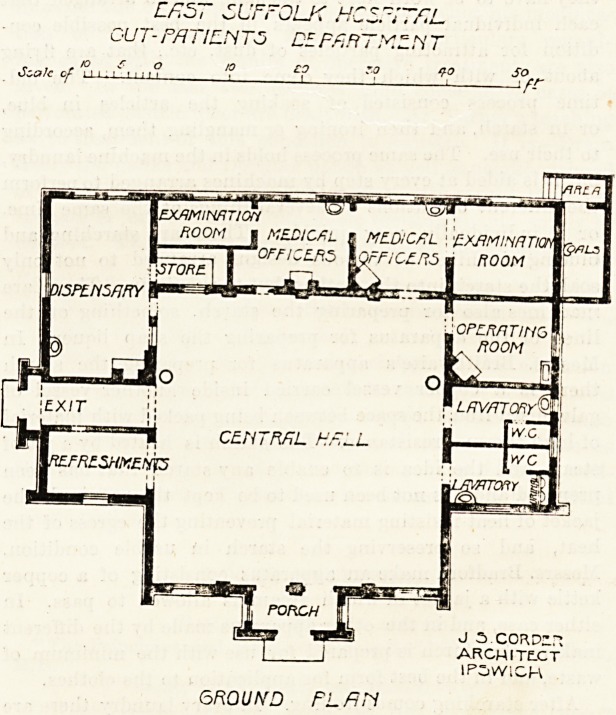# New Out-Patients' Department at the East Suffolk Hospital, Ipswich

**Published:** 1905-04-29

**Authors:** 


### NEW OUT-PATIENTS' DEPARTMENT AT THE EAST SUFFOLK HOSPITAL, IPSWICH.

This, the latest adjunct to the hospital, owes its existence to the munificence of Dr. T. H. Bartlett, of Ipswich, who has had it erected to the memory of his father, A. H. Bartlett, M.D., F.R.C.S., and it will for a long time constitute a monument in commemoration of the work of the latter, which is far more than ornamental- -it is useful.

The new building has its entrance porch to the south, and this porch leads directly into a large central hall—the out-patients' waiting-room, which is 40 feet square. This room has a large roof-light, and also two windows in the south elevation, so that it must be well lighted. On the west side of the central hall is a refreshment room, in which it is intended to supply light refreshments, and this supply will be no light boon to many a weak patient who may have a long time to wait until his turn for examination comes. It is a wonder that some provision for this supply of refreshments is not oftener found attached to our large hospitals. Next to the refreshment room is the exit lobby with its vestibules, and further to the north is the dispensary—a fine room measuring 25 feet by 14 feet. This apartment has a drug store attached to it, and there is a hatch for the delivery of medicines. Along the north front of the building are the examination-room for ophthalmic patients, the medical officers' consulting-rooms, and another examination-room. The doors of these four rooms are in line, so that for ophthalmic testing purposes it is possible to obtain a distance of 53 feet. The operation room is to the east, and close to it are two sets of lavatories and closets.

The flooring of the central hall is composed of red, hydraulic tiles, and the other rooms are laid down with maplewood blocks. Of course both of these materials are durable and nice looking, but it may be doubted whether the joints can in either case be made absolutely and permanently dust-proof. On the other hand, they are cheap and a little dust has not the same significance in the out-patients' department as it would have in the wards of the hospital. The heating and ventilating arrangements have not been lost sight of, and they are described as being "all that can be desired." The elevations of the building are in Suffolk red brick, and the roofs are covered with Broseley tiles. The architect was Mr. John Sheevell Corder, of Ipswich, and the contractors were Messrs. Catchpole and Sons.

## Figures and Tables

**Figure f1:**